# Constructing a portable optical polarimetry probe for *in-vivo* skin cancer detection

**DOI:** 10.1117/1.JBO.26.3.035001

**Published:** 2021-03-08

**Authors:** Daniel C. Louie, Lioudmila Tchvialeva, Sunil Kalia, Harvey Lui, Tim K. Lee

**Affiliations:** a University of British Columbia, School of Biomedical Engineering, Vancouver, Canada; b University of British Columbia and Vancouver Coastal Health Research Institute, Department of Dermatology and Skin Science, Vancouver, Canada; c BC Cancer, Departments of Cancer Control Research and Integrative Oncology, Vancouver, Canada

**Keywords:** polarimetry, Stokes vector, speckle, coherence length, skin cancer, low-resource setting

## Abstract

**Significance:** Management of skin cancer worldwide is often a challenge of scale, in that the number of potential cases presented outweighs the resources available to detect and treat skin cancer.

**Aim:** This project aims to develop a polarimetry probe to create an accessible skin cancer detection tool.

**Approach:** An optical probe was developed to perform bulk tissue Stokes polarimetry, a technique in which a laser of known polarization illuminates a target, and the altered polarization state of the backscattered light is measured. Typically, measuring a polarization state requires four sequential measurements with different orientations of polarization filters; however, this probe contains four spatially separated detectors to take four measurements in one shot. The probe was designed to perform at a lower cost and higher speed than conventional polarimetry methods. The probe uses photodiodes and linear and circular film polarizing filters as detectors, and a low-coherence laser diode as its illumination source. The probe design takes advantage of the statistical uniformity of the polarization speckle field formed at the detection area.

**Results:** Tests of each probe component, and the complete system put together, were performed to evaluate error and confirm the probe’s performance despite its low-cost components. This probe’s potential is demonstrated in a pilot clinical study on 71 skin lesions. The degree of polarization was found to be a factor by which malignant melanoma could be separated from other types of skin lesions.

## 1 Introduction

Skin cancer is the most common form of cancer and has had an increasing incidence in recent years.^1^ Early skin cancer detection is linked to improved prognosis. Melanoma is the deadliest type of skin cancer, but its mortality rate decreases significantly if a lesion is treated in its earliest stages.^2^ Technological assistance for skin cancer diagnosis has been an active field of research. These efforts focus on non-invasive methods, meant to assist practitioners in determining whether a lesion should be biopsied. A recent review of skin cancer detection technologies^3^ identified several key aspects that detection systems should adopt to have a high clinical impact. Examples include being low cost, compact and portable, fast, non-invasive, and having high diagnostic sensitivity. In search of this technology, tissue polarimetry has demonstrated the potential to be a strong medical diagnostic tool, conforming to the high-impact clinical aspects described above.^4^

Tissue polarimetry investigates fundamental polarization properties such as depolarization, diattenuation, and birefringence that are linked to tissue cellular and sub-cellular structural changes.^4^^–^^6^ The bulk tissue polarization characteristics could be derived from a 4×4 scattering Mueller matrix. However, Mueller polarimetry has faced challenges in clinical implementation due to large and expensive electro-optical components.^6^ The four-element Stokes vector fully describes the polarization ellipse and depolarization, though lacking quantification of birefringence and diattenuation. However, Stokes vector measurements may provide a sufficient diagnostic ability, and therefore, this technique could serve as an efficient replacement for Mueller matrix measurements. In contrast to a Mueller polarimetry system, which requires a multi-state polarization state generator (PSG) and multi-state polarization state analyzer (PSA), a Stokes polarimetry system only requires a multi-state PSA, allowing for a reduction in required measurements and acquisition time.^5^ In recent years, systems that avoid mechanical moving parts have been implemented. An apparatus using liquid crystals or photo-elastic modulators for polarization alternation can take measurements in milliseconds.^5^ However, the cost of these systems is still high.

In this paper, we report an innovative design of a fast and portable Stokes polarimetry probe that measures the depolarization of partially coherent light backscattered from skin. It is designed with simple components and intended to be easy to use in clinical settings. Polarization is measurable using low-cost films, partially coherent light is provided by simple laser diodes, and measuring backscattered light in free-space does not require lenses. The probe uses an innovative division-in-space PSA scheme to take a full Stokes vector measurement in a single snapshot. This rapid acquisition time was achieved by symmetrically placing four photodiodes with individual polarizing filters in the same detection plane to simultaneously capture the backscattering signal of an illuminating laser pulse.

The choice to use the partially coherent light of a laser diode, as opposed to noncoherent light, is motivated by an opportunity to leverage the phenomenon of polarization speckle. Speckle is only formed through coherent light scattering, and the statistical features of polarization speckle are influenced by the morphological structure and composition of an illuminated target.^7^^,^^8^ It has been found previously that the statistical moments of a polarization speckle pattern could be a useful diagnostic property for skin cancer. A previous polarization speckle-based device was able to separate melanoma versus seborrheic keratosis, a benign lesion that melanoma is often mistaken for.^9^ The average intensity of a polarization speckle pattern can be measured with a simple intensity detector such as the photodiodes that serve as this probe’s detectors.

In the operational geometry of this Stokes polarimetry probe, the backscattered laser light forms a far-field speckle pattern, which is expected to be statistically uniform over the four detectors. This is the primary novelty of our design, which allows for the replacement of a time-consuming sequential PSA with four photodiodes with film polarizers placed in front. Some testing experiments were done to confirm the workability of the new registration scheme and are reported in the sections to follow. In Sec. 2 of this paper, we review the theoretical background of polarization and speckle, to illuminate the phenomena around which we designed our polarimetric probe. Section 3 details how the probe was designed to accommodate for speckle measurement, and Sec. 4 covers the error evaluation and validation testing of the completed prototype. This probe was assessed in a preliminary clinical trial, reported in Sec. 5, involving 71 skin lesions and demonstrates the potential to separate melanoma from benign and other cancerous lesions *in-vivo*.^10^ The results of our tests indicate that this type of Stokes polarimetry probe has the qualities necessary to be an effective tool in enabling widely accessible melanoma detection.

## 2 Background

### 2.1 Stokes Vector

Polarization is the oscillation orientation of the electrical vector of a propagating light wave. This is generally an elliptical state that includes linear and circular components. Light can also be depolarized, in which the oscillations are randomly oriented. The polarization state of light can be quantified through a Stokes vector S comprising four Stokes parameters as per Eq. (1):

$$S=[\begin{array}{c} S_{0}\\ S_{1}\\ S_{2}\\ S_{3} \end{array}]=[\begin{array}{c} I_{0}+I_{90}\\ I_{0}-I_{90}\\ I_{45}-I_{135}\\ I_{RH}-I_{LH} \end{array}].$$ (1)

Each of the Stokes parameters corresponds to the difference between two orthogonal states of polarization, using a Cartesian coordinate frame of reference. $S_{0}$ is the total intensity of polarized light and can be calculated as sum of any two orthogonal components. $S_{1}$ is the difference between horizontally and vertically linear polarized components, $S_{2}$ is the difference between linear polarized components +45  deg and −45  deg from the horizontal, and $S_{3}$ is the difference in intensity between right- and left-hand circular polarized components. The Stokes parameters can be determined from six intensity measurements rather than from the sixteen measurements required form the Mueller matrix formalism. In addition, due to the relationships between the Stokes parameters, it is possible to measure a Stokes vector using only four intensity measurements. These factors make the Stokes vector a convenient object to measure. From the Stokes parameters, one can calculate the shape and orientation of the polarization ellipse and depolarization metrics.

If propagating through a turbid medium, polarized light will experience modifications on its initial state of polarization (SOP). Depolarization is the strongest of these modifications, which provides the best signal to noise ratio, as depolarization is a result of scattering, a phenomenon present in large quantities in all turbid media. Polarization is lost quickly; just several scattering events are enough to destroy the initial polarization. It is expected that the average path length of polarization preserving photons is of the order of the transport path length, which is about a few millimeters in tissue.^11^

From the Stokes vector, we can calculate depolarization metric called the degree of polarization (DOP) as in Eq. (2),^12^

$$DOP=\frac{\sqrt{S_{1}^{2}+S_{2}^{2}+S_{3}^{2}}}{S_{0}}.$$ (2)

The DOP represents the fraction of backscattered light that maintains the initial SOP. It ranges from 0 for fully depolarized to 1 for fully polarized light and could be used for tissue characterization.^8^ Values of DOP that carry information lie between 0 and 1, as the backscattered signal should be partially polarized.

While indicative of tissue optical properties, DOP measurements are also affected by the wavelength of the illuminating light source. In the same tissue sample, red light penetrates deeper and undergoes mostly volumetric scattering. In contrast, blue light has a shallower penetration and carries mostly surface information. The difference in illuminated tissue volume affects DOP due to the difference in cellular composition of those volumes.

### 2.2 Speckle

While noncoherent light can be used for polarimetry, we have opted to use partially coherent light to take advantage of the modification of wave phase-encoded in speckle. When coherent or partially coherent light is scattered it generates speckle, a stochastic interference pattern in the form of dark and bright spots. Speckle patterns are composed of numerous speckles that can each have an individual size and shape, intensity, and SOP.^13^ Each speckle is the result of the interference of stochastically distributed light of similar SOPs. The SOP and corresponding Stokes vector may vary from speckle to speckle and in certain scattering conditions, the polarization states among the speckles become non-uniform, forming a phenomenon called polarization speckle.^14^^,^^15^ The composition of polarization speckle is highly sensitive to the phase of light, and thus speckle patterns generated from illuminating tissue can contain additional valuable information on tissue morphology. For example, speckle has been found useful in measuring skin surface roughness, with applications in skin cancer detection.^9^

### 2.3 Polarization Speckle Design Considerations

We have two primary design considerations in measuring polarization speckle. The first consideration is ensuring the development of an informative polarization speckle field. Polarization and coherence are mutually connected,^16^ and light depolarization occurs at comparable propagating distances to light decorrelation (the decay of polarization matching the decay of coherence). As mentioned previously, only DOP measurements between 0 and 1, convey tissue information. Therefore, emerging light is preferably partially polarized and correspondingly partially coherent. A partially coherent speckle pattern is produced when the average photon path length difference in tissue is on the order of the temporal coherence length of the illuminating source.^17^ If the path length difference is too large, then the returning light will fail to be coherent and a pattern will not appear. To ensure partially polarized backscattered light, we require a laser with a coherence length on the order of millimeters to match the expected depolarization length.^11^ This is one of the main features that influenced the choice of laser in the design of this probe, as expanded upon in Sec. 3.2.

The second consideration is the validity of spatial averaging of polarization within a speckle field. Since each speckle will maintain its own Stokes vector, and the SOPs would be distributed stochastically over the detection area, then spatially averaging a measurement across an increasing number of speckles may force measured DOP toward 0.^14^ We can adjust the detection geometry to ensure an appropriate number of speckles appear on the detector area. Essentially, the number of speckles on the detector must be large enough to ensure accurate mean intensity measurements, but not too large such that the stokes parameters are artificially reduced due to spatial averaging. In this article, we present two studies for this design consideration: calculating the proper relationship between detector size and the distance between object and detector, and ensuring the detection of a uniform field. The first of these studies is listed in Sec. 3.3. The probe’s response to field uniformity is detailed in Sec. 4.2.

## 3 Component Design Considerations

### 3.1 Polarizing Filters

Mathematically, it is possible to determine the Stokes vector of light using any four intensity measurements taken from unique polarization states on the surface of the Poincaré sphere, a geometrical model of polarization. In practice, this appears in division of focal plane polarimetry methods that measure polarization with four separate detectors in space, with devices such as pixel polarization cameras. Studies in this area have noted that systematic error decreases if the measurement points around the Poincaré sphere are equidistant in space.^18^ However, our probe is designed with the simplest configuration to implement; that of four identical film circular polarizing filters in four independent rotation angles. This scheme takes advantage of the assumption that the sum of intensities measured from two orthogonal polarizing analyzer orientations (any two perpendicular linear polarizations, or left- and right-hand circular polarizations) are the same regardless of which two orientations they are. The full details of this scheme can be found in our previous work.^10^ This is a resource-efficient scheme first described by c^19^ that requires only one type of polarization film, a circular polarizer comprising a linear polarizer and quarter-wave plate, as opposed to four unique elliptical filters.

To set the laser diode’s initial polarization to match the device detectors’ axes, a polarizing filter is placed before the laser. This is a wire grid linear polarizing filter (ThorLabs, WPL12-VIS). When the device is set for initial circular polarization, this is followed by a film circular polarizing filter. To note, this is the same film used as analyzers for the detector; an intentional choice for resource efficiency.

### 3.2 Laser Diode

We selected a laser diode similar in wavelength to ones previously used for speckle work (660 nm, 120 mW, Thorlabs, HL6545MG).^7^ This laser diode was tested through Michaelson interferometry to determine if the coherence length of our selected laser diode is on the order of the path length that the light would travel through tissue. The temporal coherence length was found to be ∼3000  μm, which is on the order of the photon path length for red light in skin, and therefore an appropriate length for speckle generation.

According to the American National Standard for Safe Use of Lasers,^20^ the maximum permissible exposure (MPE) for skin exposure to a laser beam for durations between $10^{-7}$ and 10 s is described by the equation

$$MPE=1.1*C_{A}*t^{0.25},$$ (3)

where $C_{A}$ for 400- to 700-nm wavelength light is 1, t is exposure time in seconds, and MPE is in units of $J/{\mathrm{cm}}^{2}$. The collimation package for the laser diode (Thorlabs LT230P-B) has a minimum laser beam diameter of 0.5 mm. Although it is larger in practice (measured to be 3 mm), we performed the safety calculation to accommodate for the minimum area. As measured, the incident power of the laser on the skin is recued to 67 mW by the initial linear polarizer, however, we performed this calculation at 120 mW to allow for a margin of error. The maximum exposure time is be calculated to be 4.7 ms, which is our final laser pulse duration.

### 3.3 Device Geometry

Several aspects of the probe’s geometry address polarization speckle. First, we must ensure that the speckle field is statistically uniform at the device’s detection plane, which requires a detection plane in the far diffraction zone. For speckle, it was shown that, instead of the common formula, the distance z between the detector and the object for which the far zone occurs obeys the condition in Eq. (4):^21^

$$z\gg \frac{q^{2}}{0.664L_{c}},$$ (4)

where q is the diameter of backscattered light from the skin, and $L_{C}$ is the temporal coherence length (3 mm). The diameter of backscattered light will vary depending on skin conditions in the range of 3 to 7 mm, decreasing for more absorptive targets, but never to less than the measured beam diameter of 3 mm. In non-lesion fair skin, the backscattered light diameter was measured to be ∼7  mm, which an approximate maximum size we will use for this calculation. For this prototype, we have a low-coherence diode laser with an $L_{C}$ and beam diameter both on the order of millimeters, therefore the far-field zone z is achieved with a height >2.5  cm. This is easily attainable in a handheld device.

**Fig. 1:**
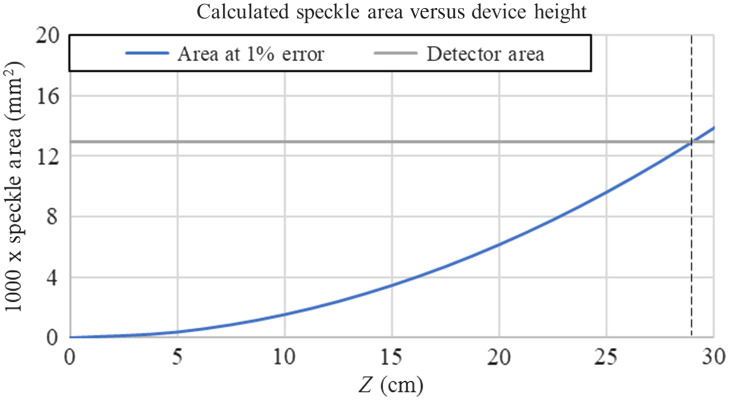
Plotting speckle area versus device height. The horizontal line indicates $13\;{\mathrm{mm}}^{2}$, the size of our photodiodes.

The second aspect is how to limit the amount of speckles N averaged over the detection area while ensuring a sufficient amount are detected to reduce statistical error. In free-space geometry the number of speckles on a detector can be evaluated as in Eq. (5),^22^

$$N=\frac{A_{D}}{A_{S}},\;A_{S}=\frac{{\left(\lambda z\right)}^{2}}{\pi q^{2}},$$ (5)

where $A_{D}$ is the detector area, $A_{S}$ is the spatial coherence area (or speckle size), λ is the laser wavelength (660 nm), z is the distance between object and detector (to be determined), and q is the backscattered spot diameter (ranging from 3 to 7 mm). As speckles form a stochastic field, any statistical observation of the field, such as an intensity average, can be subject to sampling error due to observing too few speckles. The number of speckles N should be large enough to reduce the error of an average intensity measurement while being low enough to not create added depolarization due to spatial averaging on the detector area. This error is on the order of the inverse square root of N.^23^ Our chosen error threshold is 1%, which would require ∼10,000 speckles on our detector area.^23^ In this prototype, our detectors are photodiodes (Thorlabs, FDS100) with a $3.6\times 3.6\;{\mathrm{mm}}^{2}$ detector area ($13\;{\mathrm{mm}}^{2}$). From this, we can observe that we must select the height of our device to ensure a speckle area less than 10,000 times smaller than the detector area. As a 3-mm backscattered light diameter would result in the fewest generated speckles, we will determine our maximum size based on this measurement. Plotting 1000× speckle area versus device height as seen in Fig. 1 generates a quadratic function. The intersection of 1000× speckle area and our detector area reveals the range of applicable device heights, where the theoretical maximum is just below 30 cm. This maximum increases for larger spot sizes, leaving this estimate of 30 cm as an appropriate boundary.

For final device height, we are given a functional range between 2.5 and 30 cm. This range also works well with other restrictions that device height would generate. As we are observing backscattered light, the intensity of the signal decreases with the inverse-square of the distance. Secondly, a smaller height would make the device more portable, and a probe with a length greater than 20 cm would be unwieldy.

**Fig. 2:**
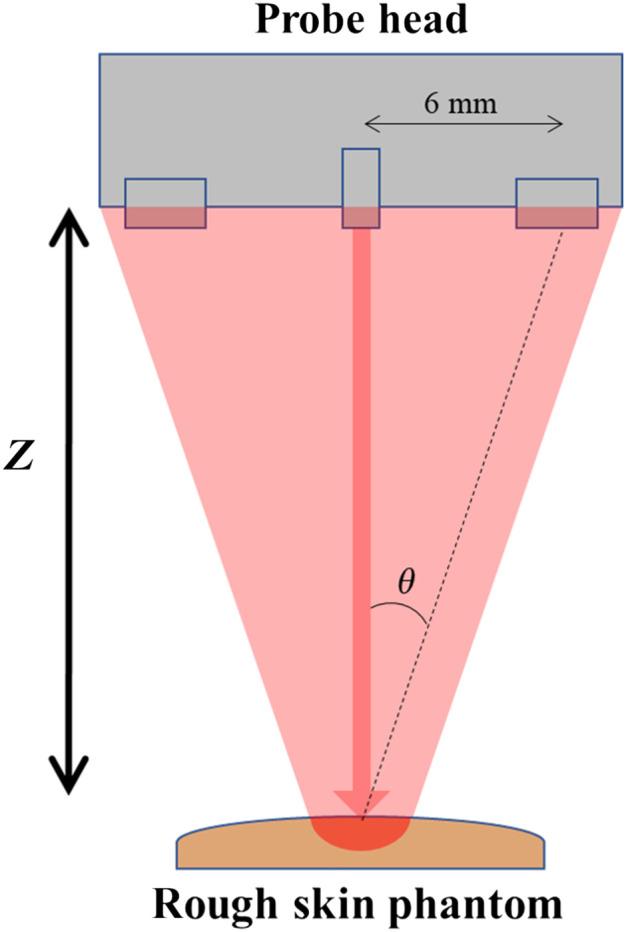
Diagram of device height testing.

**Fig. 3:**
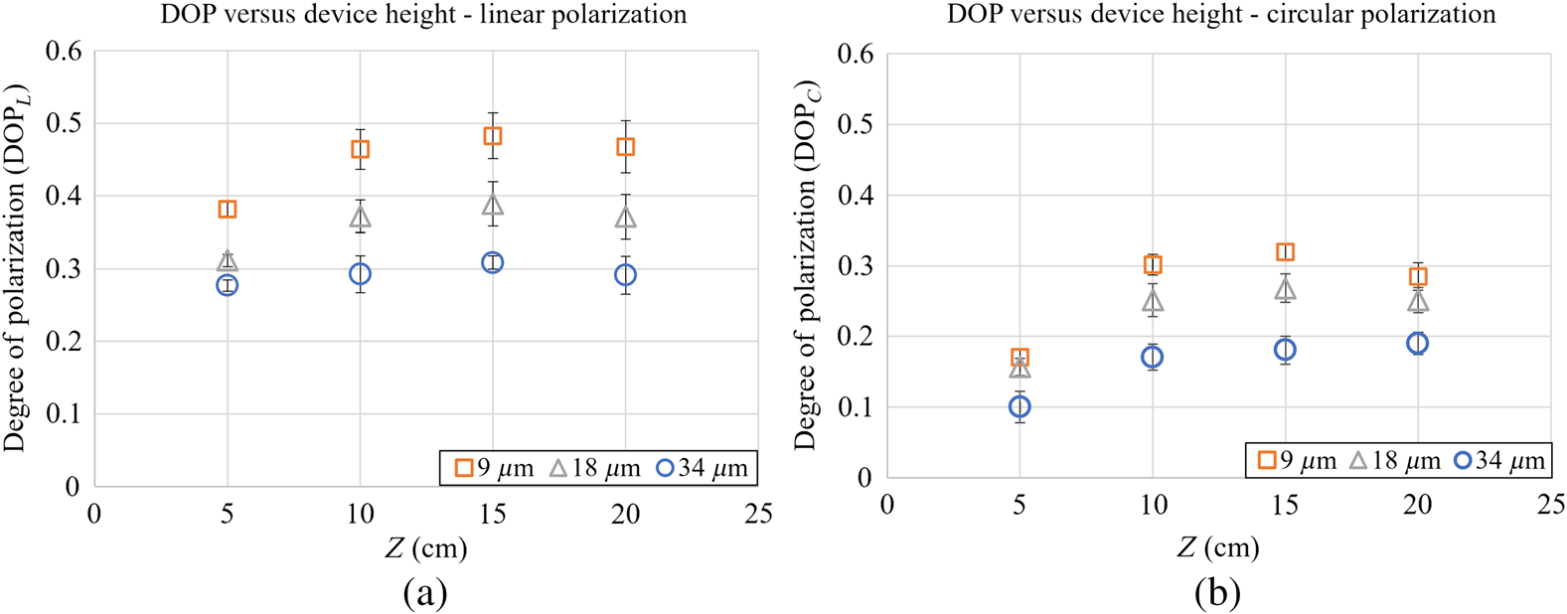
(a) DOP versus device height for initial linear and (b) circular polarizations. Measurements shown on three different RMS surface roughnesses (9, 18, and 34  μm). Error bars indicate standard deviation of 10 measurements.

A series of tests were done to determine the effect of the probe’s height on its measurements. These tests were carried out validate our theoretical model of polarization speckle development and test for the appropriate distance (Z) for the device. DOP measurements using both linear (${\mathrm{DOP}}_{L}$) and circular (${\mathrm{DOP}}_{C}$) initial polarizations were taken using the probe at heights of 5, 10, 15, and 20 cm over different RMS roughnesses of silicone skin phantoms (heights chosen to sample from a practical range). The properties of these phantoms have been previous reported,^10^ along with their recipe.^24^ The matrix of the phantoms was made from Smooth-On brand MoldMax 10T silicone and was pigmented to resemble human skin tones with a combination of Silc-pig silicone pigments (Smooth-On, Inc.). This silicone begins as a viscous resin, and is mixed with a curing agent at room temperature to hold its form. The silicone was molded with a metal roughness standard (Microshurf #334 comparator, Rubert+Co Ltd., Cheadle). The phantom roughnesses were verified by a WYKO NT2000 optical profilometer (Veeco, Tucson, Arizona). The testing scheme is shown in Fig. 2 and the results are in the following Fig. 3. For each measurement in Fig. 3, 10 measurements were taken and averaged from points on the corresponding roughness area of the phantom. Error bars indicate the standard deviation of measurements.

Three tendencies can be observed. First, the DOP for circularly polarized light is consistently lower than the DOP for linearly polarized light, which implies that the skin phantoms are in the Rayleigh regime of depolarization. Second, DOP is smaller for rougher surfaces, demonstrating that roughness introduces additional depolarization.^25^^,^^26^

The third observation is that for both modes of input light and all roughnesses, the DOP increases from 5 to 10 cm but saturates beyond 10 cm. The decrease in DOP from 10 to 5 cm is explainable as in decreasing Z we approach a Fresnel zone of diffraction.^8^ This would cause the path difference between central and peripheral rays to become larger and exceed the temporal coherence length, leading to polarization and light coherence decay. After 10 cm, since the speckle area increases while the detector area remains constant, the number of speckles on the detector is decreasing, but the average polarization and their metrics such as DOP could stay the same if the speckle field is statistically stationary. The complete extent of DOP versus roughness versus device height is still subject to further study, but recognizing the additional preference for a lower height to increase signal strength, we chose a final height of 10 cm.

### 3.4 Completed Prototype Design

**Fig. 4:**
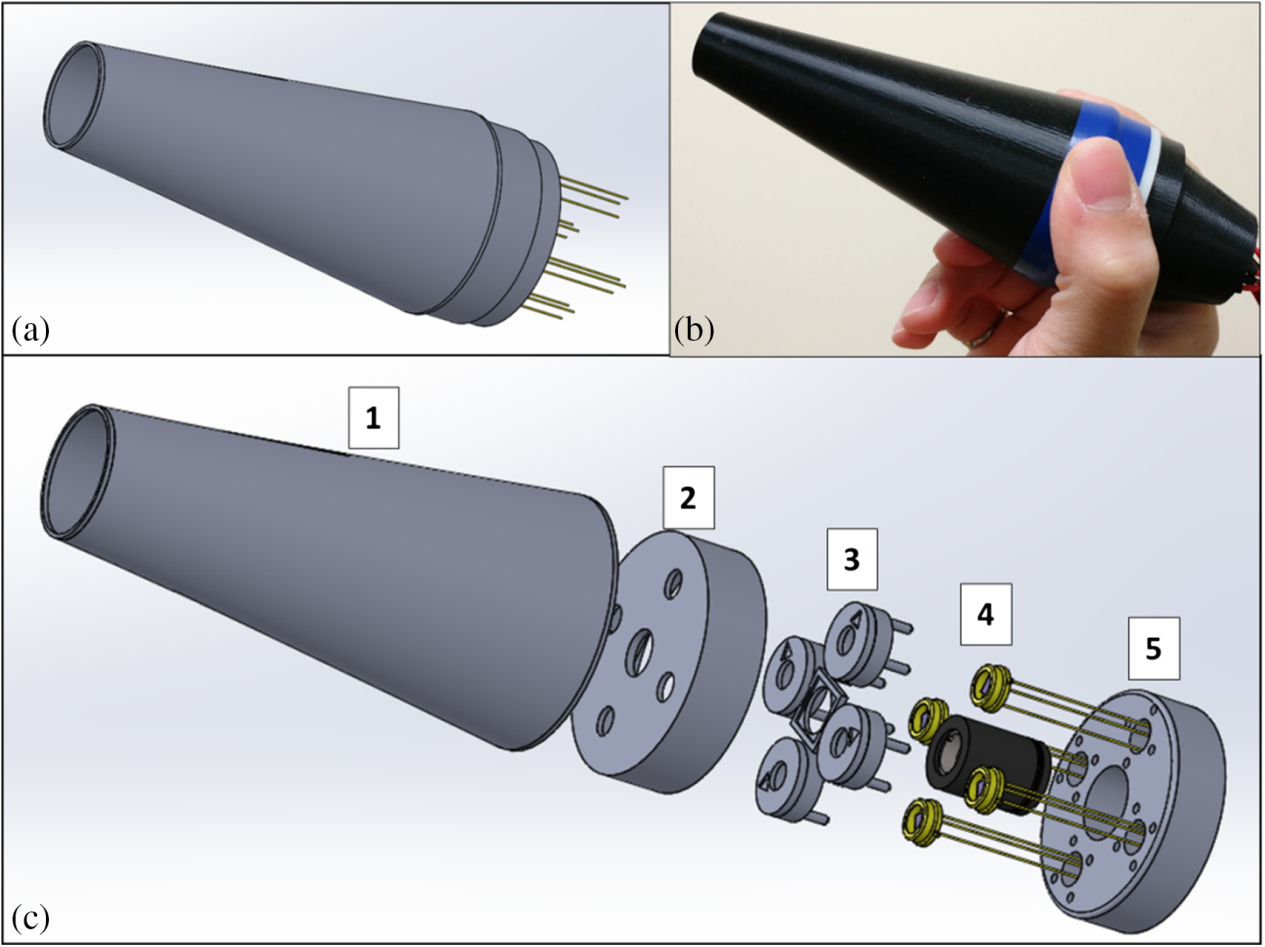
(a) Model of the Stokes polarimetry probe. (b) Photo of the probe held in hand. (c) Expanded view of probe components. Includes (1) targeting cone, (2) and (5) casing, (3) components to hold polarizing film, and (4) laser diode and photodiodes.

Figure 4 shows a model and photo of the probe head, and a view of its internal structure to illustrate the major functional blocks. At this prototype stage of development, the probe head does not contain purely electronic components. These are kept in a connected casing that is also connected to a computer. The probe’s measurements are recorded on this computer through a LabVIEW UI. In a more developed prototype, the electronic and computation hardware could be minimized in both component cost and size. The radial distance at which the photodiodes are placed is determined by two design constraints; the first being the device’s handheld size, and the second being the acceptance angle (15 deg) of the polarizing filters used. As shown in Fig. 2, the photodiodes are 6 mm from the laser diode at the center, such that an angle within the film’s acceptance (θ=4  deg<15  deg) is observed between the center of the photodiode and the incident site. The outer dimensions of the probe head are 12.5 cm in length and 5 cm in diameter.

The tested prototype is primarily composed of fitted and pressed 3D-printed components. The inside of the targeting cone shown in Fig. 4 (1) is coated with optically dampening velvet to eliminate ambient reflections. To aim the probe, a sticker is applied centered on the target site within which the targeting cone fits. These stickers are a standardized and common landmarking tool in dermatology, to assist in labeling sites of interest and observing their scale in photographs. With the press of a button (not shown in the figure) the laser diode (4) is fired, passing through an input polarizer (3), and striking the target. The backscattered light is observed equally by the photodiodes (4) through their respective polarizing filters (3), which are all held in the same plane to allow for uniform speckle field distribution.

## 4 Measurement Testing and Validation

To examine this device’s ability to measure the Stokes parameters in one shot, two tests were devised to measure errors in the system. The first is a test of the circular polarizing film, to examine the polarization state of initial light. The second is a test of the probe’s measurement abilities incorporating all four detectors, to determine the device’s ability to measure the Stokes parameters of a uniform field. Both these aspects, light generation and the geometric scheme for measurement, are crucial to validate the components and the design. Following these tests, a pilot clinical trial was performed to evaluate the probe’s performance in the field.

### 4.1 Polarized Light Generation—Circular Polarizing Film

The circular polarizing film (Bolder Vision Optik) was specified at a center wavelength of 660 nm. This film is a laminated combination of linear polarizer and quarter-wave plate. The first test we performed is the analysis of this film in conjunction with the laser diode as a circular PSG. While the film was made to tight specifications, a laser diode is not perfectly monochromatic, which likely reduces the film’s effectiveness by some margin. For this test, laser light was passed through this circular polarizing film. A wire-grid linear polarizer was placed in the optical path as an analyzer and was rotated as transmission was measured.

**Fig. 5:**
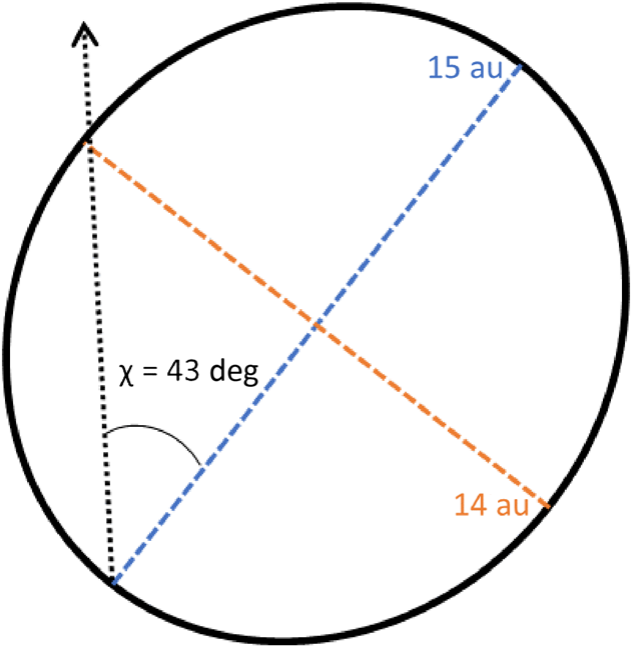
Diagram of polarization ellipse of the probe’s initial elliptically polarized light.

By observing the fluctuation in intensity between these two angles of the linear polarizer, we can determine the ratio of the two arms of the beam’s polarization ellipse, which informs us of the probe’s initial polarization state. For perfectly circular polarized light, there should be no fluctuation in intensity. In the presence of ellipticity, the measurement will be different at the angles perpendicular and parallel to the ellipse’s major axis, as shown in Fig. 5. We measured an intensity ratio difference of 15:14. Computing the ellipticity angle from the ratio results in χ=43  deg. When being used as part of a circular PSG, the resulting normalized $S_{3}=\sin(2\chi )=0.998$, with a corresponding linear component of $S_{1}=\cos(2\chi )=0.068$. It is worth noting that the linear component is represented by both $S_{1}$ and $S_{2}$, but in this experiment the azimuthal angle of the ellipse was arbitrary, so we model the entire linear component using $S_{1}$. The resulting normalized polarizance vector of the circular polarizing film is ${\left[1,0.068,0,0.998\right]}^{T}$, which makes the light slightly elliptical.

### 4.2 Polarized Light Measurement—Error Evaluation

**Fig. 6:**
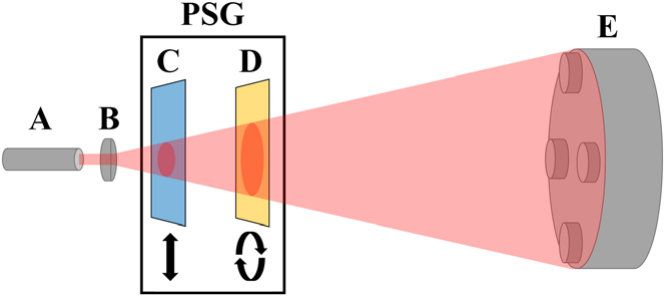
Diagram of field uniformity test with depiction of expanded laser light. (A) Laser diode. (B) Magnifying lens. (C) Rotatable linear polarizer. (D) Non-rotating quarter-wave plate. (E) Probe head.

Once the probe’s detection scheme was assembled, we simulated polarized backscattered light to evaluate the error present in the system. These experiments were performed using the framework show in Fig. 6. The probe head (E) was centered within the beam originating from (A) and expanded using a magnifying lens (B). Following this, adding a rotatable linear polarizer (C) allows the beam to be swept through all angles of linear polarization. By placing a fixed quarter-wave plate after the linear polarizer (D), we can also sweep through degrees of elliptical polarization. To note, this process created monochromatic polarized light but not polarization speckle. The intensity readings of the four photodiodes were recorded, and then combined into a Stokes vector, normalized such that the first element $S_{0}$ is 1.

**Fig. 7:**
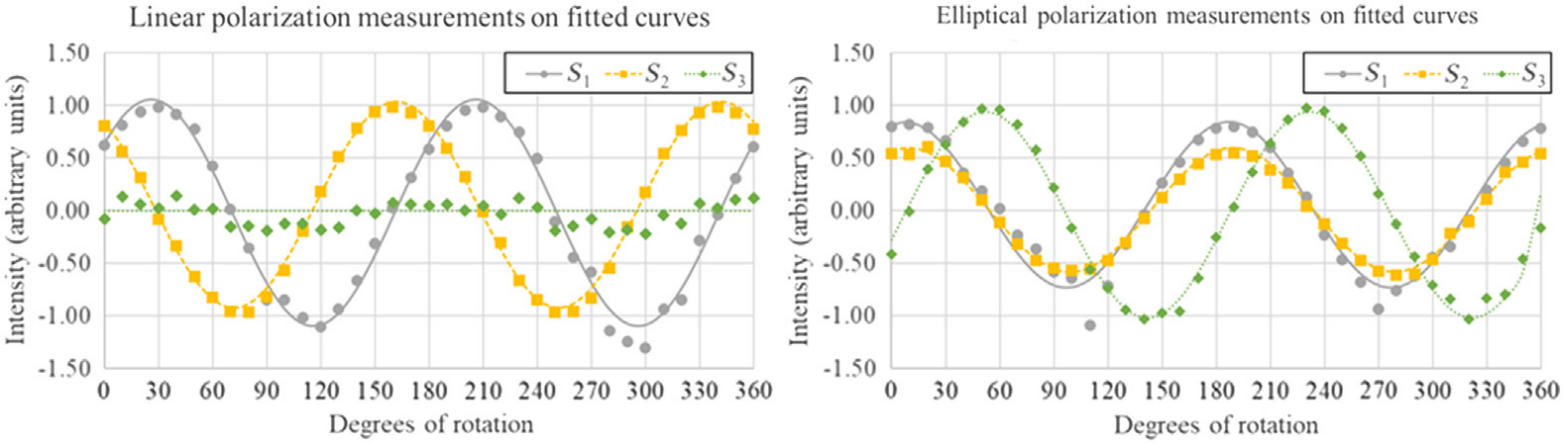
Linear and elliptical polarization measurements compared against fitted sine curves.

Measuring the Stokes parameters while sweeping through all angles of linear polarization and degrees of elliptical polarization would generate a sinusoidal function. For a perfect polarimeter tracking a linear sweep, the measurements of $S_{1}$ and $S_{2}$ would perfectly fit a sine function, and $S_{3}$ would read a constant 0. For an elliptical sweep, all three Stokes parameters would fit a sinusoidal function. To evaluate the error of these measurements, we fitted each of our measurements to a sine function (or with 0 in the case of linear sweep $S_{3}$) and calculated the RMS error, assuming that the fitted function approximates the ideal. In each case, the Stokes parameters were normalized by dividing by $S_{0}$ (which represents intensity), such that $S_{0}=1$. These fittings are shown in Fig. 7. In addition, we calculated the RMS error of our measured DOPs for each measurement against an ideal DOP of 1. In this testing scheme, the DOP of every state should be close to 1, as this is a direct polarization measurement without scattering or speckle generation. These results are reported in Table 1.

**Table 1:** Total RMS error analysis.

Fitting to sine function:		
Linear sweep		Absolute RMS error
	S1	0.09
	S2	0.05
	S3	0.12
Elliptical sweep		Absolute RMS error
	S1	0.12
	S2	0.03
	S3	0.08
Absolute RMS error in DOP		0.10

From these tests, we can also investigate the sources of error for this probe. We measured the variance in measurements when presented with the same depolarized light over time. This allows us to approximate the random error present in our measurements, separate from the systematic error caused by our PSA scheme and ellipticity of our filters. We found that by propagating error from the photodiode measurements up to DOP calculation, the final random error is ∼0.02.

This finding indicates that most of our error is generated by systematic sources, which is also observable in the data from the sweeps. The measured points become more distant from the curve at specific angles in the sweep, and in fact, the Stokes parameters exceed a magnitude of 1 at those angles, which should be an impossible value. These values occur due to the Stokes parameter calculations relying on the measurements close to the extinction angle of the polarizing filters, where photodiode sensitivity is greatly reduced. Errors caused by non-uniformity of the field across the detector face will be amplified at these angles, which can result in calculating Stokes parameters with absolute values >1. It is anticipated that this error could be reduced by selecting a different PSA filter scheme, as expanded upon below.

The errors present in this probe can be considered in the greater context of Mueller matrix polarimeter error analysis. As reported by Tyo,^27^ the error inherent to an PSG or PSA can be calculated from their characteristic matrix, a matrix formed from their polarizance vectors. To reduce error, the matrix must be well-conditioned, a property that indicates a small change in output when provided with a small change in input. This is observed mathematically through the matrix’s condition number, a value calculated from the ratio of the largest and smallest singular values of the characteristic matrix. This condition number spans the range of 1 to infinity, where 1 indicates perfect conditioning. Our PSA’s characteristic matrix is in Eq. (6):

$$P^{\det}=[\begin{array}{cccc} 1 & 1 & 1 & 1\\ 1 & 0 & -1 & 0\\ 0 & 1 & 0 & 0\\ 0 & 0 & 0 & 1 \end{array}].$$ (6)

More detail of this calculation can be found in established work.^27^^–^^29^ From our PSA, we can calculate a condition number of ∼3.2, which is greater than Tyo’s calculated minimum possible condition number of $\sqrt{3}$. Bruce et al.^28^ experimentally determined the error present in Mueller matrix polarimeters of various condition numbers. It was estimated that a Mueller matrix polarimeter with a condition number of ∼3.2 would see 10% RMS error (0.1 for a normalized Mueller matrix) if the signal is subject to a noise level of 5%. While we expect that the error of a Stokes vector polarimeter would be less than that of a Mueller matrix polarimeter due to the fewer number of measurements required, this estimate corresponds to the experimental error of our findings. As these estimates match, we consider our results to indicate good performance given the lesser robustness of this probe relative to standard polarimetric devices.

## 5 Pilot Clinical Trial

To test this probe’s ability in a clinical setting, a pilot clinical study of 71 skin lesions across 49 patients was performed using the probe. This trial is reported more in-depth in a previously published paper on clinical usefulness of the DOP metric.^10^ A brief summary will be presented here for completeness. The lesions tested in this study included three types of skin cancer and three types of non-cancerous lesions: malignant melanoma (MM), basal cell carcinoma (BCC), squamous cell carcinoma (SCC), melanocytic nevus, SK, and actinic keratosis, respectively. Diagnostic tools are required to improve the clinical diagnostic accuracy to distinguish skin cancer lesions within this set. Melanocytic nevi measured for this study were deemed suspicious for cancer and were confirmed benign after a biopsy. Similarly, all cancerous lesions had their final diagnosis performed through a biopsy and histological examination. Only three of the lesions had different clinical and pathological diagnoses.

**Fig. 8:**
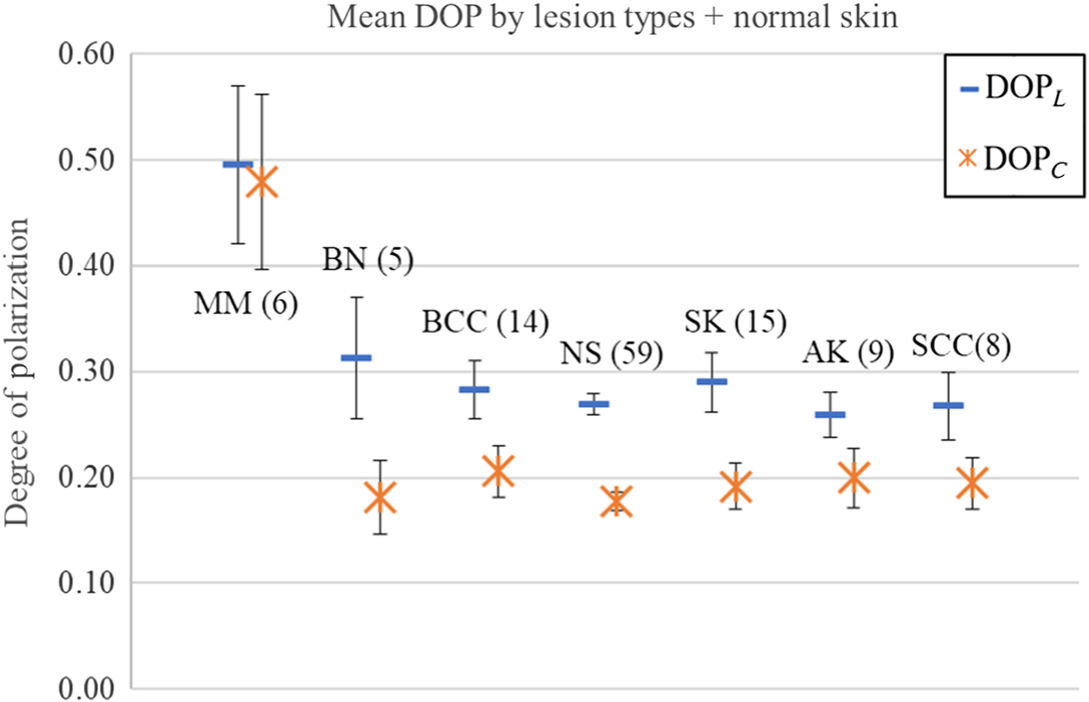
Mean DOP by lesion types, including NS. Data found in Table 2.

During data analysis, some measurements were found to display suspicious or erroneous values. These included data points where the Stokes parameters were of absolute value far greater than 1, resulting in outlying DOP values. These types of invalid measurements were found to be caused by difficulties in aligning the probe with skin lesions on irregular skin sites such as the nose and ear. Should the probe be too far from perpendicular with the measurement site, the backscattered light fails to be a uniform field, resulting in some detectors receiving more illumination than others. Another type of error observed is a mismatch in DOP and the input polarization. Since tissue is a primarily depolarizing medium, it is expected that for linearly polarized input light the degree of linear polarization (DOLP) of backscattered light should still exceed the degree of circular polarization (DOCP), and vice-versa for circular input polarization. In cases where the reverse is observed (linear input but DOCP > DOLP or circular input but DOLP > DOCP), this could also be due to misalignment or even human error in labeling which input polarization was used. These cases overlapped with the irregular body site restriction and were similarly removed. Future improvements on this device scheme will address these problems and simplify obtaining measurements from these body sites. After eliminating these measurements, 61 valid target sites remained split among 57 lesions and 59 normal skin (NS) sites. Lesions are distributed as in Table 2 and shown in Fig. 8. The standard error is reported to compare the mean measurements for each lesion type. The lesion types are listed in order of approximate roughness from smoothest to roughest, as determined by previous speckle studies and clinical experience. As an additional note, absorption has a known influence on DOP, with highly absorbing media displaying increases in DOP. This is because the observable backscattered photons come from shallow layers of the medium, where they undergo fewer scattering events compared to the depolarized photons, which penetrate deeper and have a lower probability to escape.^30^^,^^31^ Melanoma lesions are typically the darkest among the presented lesion types, with benign nevus (BN) also being similarly dark. Garcia-Uribe et. al.^32^ has measured the *in-vivo* average absorption coefficient of melanoma to be $0.9\;{\mathrm{cm}}^{-1}$, and nevus to be $0.8\;{\mathrm{cm}}^{-1}$ at our probing wavelength of 660 nm. BCC, SCC, and actinic keratosis have lower average absorption coefficients at 0.6, 0.5, and $0.4\;{\mathrm{cm}}^{-1}$, respectively. Lesion pigmentation was not explicitly monitored for this study, though the metric could be quantified with a colorimeter for closer analysis of the relationship between absorption and DOP in a closer examination.

**Table 2:** Summary of target types and DOP in clinical trial.

Lesion type	Diagnosis	N	Mean ${\mathrm{DOP}}_{L}$ a.u. (std err)	Mean ${\mathrm{DOP}}_{C}$ a.u. (std err)
MM	Cancer	6	0.50 (0.07)	0.48 (0.08)
BN	Non-cancer	5	0.31 (0.06)	0.18 (0.03)
BCC	Cancer	14	0.28 (0.03)	0.21 (0.02)
NS	NS	59	0.27 (0.01)	0.19 (0.01)
SK	Non-cancer	15	0.29 (0.03)	0.19 (0.02)
SCC	Cancer	8	0.26 (0.03)	0.19 (0.02)
Actinic keratosis	Pre-cancer	9	0.26 (0.02)	0.20 (0.03)

The DOP was calculated from the measured Stokes vectors using both linear initial polarized light (${\mathrm{DOP}}_{L}$) and circular initial polarized light (${\mathrm{DOP}}_{C}$). Recognizing that a small sample size decreases our ability to make statistical inferences, the measured mean ${\mathrm{DOP}}_{C}$ for ${\mathrm{MM}}_{C}$ (0.48±0.08) stands separate from ${\mathrm{BN}}_{C}$ (0.18±0.03). Similarly, the measured mean ${\mathrm{DOP}}_{L}$ for ${\mathrm{MM}}_{L}$ (0.50±0.07) and ${\mathrm{BN}}_{L}$ (0.31±0.06) have a small difference that could become more pronounced with a larger sample size. This separation indicates the potential clinical effectiveness of a probe applying our polarization speckle technique using low-cost components.

## 6 Discussion and Conclusion

With the aim of developing an accessible skin cancer detection technology, polarization speckle was identified as a promising technology. We evaluated the physical properties and potential constraints that would guide the construction of a portable low-cost polarization speckle probe, including the generation and detection of a uniform partially polarized speckle field. A Stokes polarimetry probe was constructed using low-cost components, guided by this process.

We have performed a variety of tests to determine the performance of low-cost components used in the probe and the probe as a whole. The presented laser diode, photodiodes, and polarizing film demonstrate expected performance in a laboratory setting and show promising results in a clinical trial. The error in the probe’s DOP measurements is about 10%, which is a reasonable amount of error given the simplicity of the probe’s optical schemes and the prototype nature of its design. Further testing with a more robust iteration of this device could be done to separate error due to components and construction and due to the PSA itself for a more specific evaluation. In addition, the specific factors that cause error within the unique geometry of this device would require further understanding of rapid polarization speckle measurement. The visualization of a full-Stokes polarization speckle field would answer many of these questions, such as the uniformity of individual SOPs within a speckle pattern and the degree to which increasing speckle numbers affect DOP. These will be the subject of our future investigations.

Finally, not only is the probe itself low-cost, but the equipment used to test this probe in all stages was done with simple polarization equipment, feasible to perform in a low-resource setting. With this testing, we have determined that this application of polarization speckle in the technique of one-shot Stokes polarimetry can provide useful diagnostic measurements and is implementable as a portable low-cost probe.
